# Oxygenation Indices with Oxygen Challenge Test in Neonates During VA ECMO

**DOI:** 10.3390/children13081107

**Published:** 2026-08-19

**Authors:** Abhinav Totapally, Camila De Avila, Keith Meyer, Lian Santiago, Felipe Pedroso, Fuad Alkhoury, Balagangadhar R. Totapally

**Affiliations:** 1 Division of Critical Care Medicine, Nicklaus Children’s Hospital, Miami, FL 33155, USA; camiladeavilamd@gmail.com (C.D.A.); keith.meyer@nicklaushealth.org (K.M.); balagangadhar.totapally@nicklaushealth.org (B.R.T.); 2 Herbert Wertheim College of Medicine, Florida International University, Miami, FL 33155, USA; 3 Department of Pediatric Surgery, Nicklaus Children’s Hospital, Miami, FL 33155, USA

**Keywords:** venoarterial extracorporeal membrane oxygenation, newborn, respiratory insufficiency, shock, physiology

## Abstract

**Highlights:**

**What are the main findings?**

- Oxygenation indices measured during the oxygen challenge test (OCT) on VA ECMO did not distinguish survivors from non-survivors.
- Oxygenation indices measured after decannulation, particularly shunt fraction and P/F ratio, demonstrated good discrimination for hospital mortality.

**What are the implications of the main findings?**

- The oxygen challenge test alone should not be relied upon to predict survival, and its use for readiness for decannulation during neonatal VA ECMO warrants further evaluation.
- Oxygenation assessment during trial-off or after decannulation may provide more clinically useful information for predicting outcomes and guiding ECMO weaning strategies.

**Abstract:**

**Objective:** To evaluate the response of various oxygenation indices to the oxygen challenge test (OCT), prior to decannulation, in neonates supported with VA ECMO for respiratory indications, and to compare these responses between survivors and non-survivors. **Design:** Single-center retrospective observational study. **Setting:** Single, tertiary care, 40-bed Pediatric Intensive Care Unit in Miami, Florida. **Patients:** Neonates cannulated to VA ECMO for respiratory failure from 2012 to 2022 who had an OCT. Patients with congenital heart disease were excluded. **Measurements and Main Results:** A total of 63 neonates were included, of whom 13 patients died (20.6%). Oxygenation indices compared before and during OCT include PaO2, Delta PaO2, P/F ratios, Alveolar-arterial gradient, arterial/alveolar ratios, and shunt fraction. The Wilcoxon signed-rank test demonstrated increases in all oxygenation indices during OCT compared to pre-OCT, except for P/F ratios. There were no differences in oxygenation indices between survivors and non-survivors during OCT. However, after decannulation, all oxygenation indices were worse in non-survivors (*p* < 0.05). Linear regression analysis demonstrated that delta FiO2 on the ventilator, P/F before OCT, and ECMO flow during OCT significantly affected PaO2 response during OCT. Shunt fraction above 28.8% after decannulation demonstrated the highest discriminatory ability for mortality (AUC 0.853). **Conclusions:** The OCT response during ECMO is not a reliable predictor of survival in neonates on VA ECMO, and its value in assessing readiness for ECMO trial-off warrants further evaluation. Further studies investigating the role of oxygenation indices at the time of trial-off for predicting outcomes may be helpful.

## 1. Introduction

The Extracorporeal Life Support Organization (ELSO) reported over 20,000 neonates were supported on extracorporeal membrane oxygenation (ECMO) during 2009 to 2022, with a steady yearly number [1]. The survival for neonates with respiratory indications during this period was 69% [1]. Common indications for neonatal respiratory ECMO support include persistent pulmonary hypertension of the newborn (PPHN), congenital diaphragmatic hernia (CDH), meconium aspiration syndrome (MAS), and sepsis [2,3]. ECMO support can be beneficial in these patients, especially when initiated early. While established guidelines define the initiation criteria for ECMO, the indications, weaning process, and subsequent decannulation are not well established [4,5,6]. Weaning from ECMO support is indicated when cardiac and pulmonary functions have recovered. Assessment of readiness to wean is crucial for identifying those patients who may no longer require or benefit from ECMO, thereby decreasing the risk of adverse effects associated with ECMO. Estimating readiness to wean requires objective measures to determine whether innate lung function has improved sufficiently to support the gas exchange required to meet the patient’s metabolic demands.

The oxygen challenge test (OCT) or hyperoxia test, also known as the Cilley test, is one of the tests used to assess lung function in oxygen transfer [6,7]. This test has been shown to be beneficial in patients on veno-venous (VV) ECMO for assessing lung function recovery [6,7]. This test is done by increasing the FiO_2_ on the ventilator to 100% and subsequently obtaining an arterial blood gas to evaluate the oxygenation response. After the assessment is completed, the FiO_2_ is then returned to the previous level [6]. The magnitude of rise in PaO_2_ with increasing FiO_2_ depends on the amount of venous admixture or shunt fraction [8]. The relationship between FiO_2_ and PaO_2_ depends on the pulmonary shunt (Qs/Qt), the arteriovenous oxygen content gradient, hemoglobin concentration, PaCO_2_, and displacement of the oxygen-dissociation curve, the last three being of relatively minor importance [9,10,11]. Iso-shunt curves were developed to calculate the desired FiO_2_ based on the FiO_2_ and PaO_2_ response. The OCT is based on this physiologic principle that the increase in PaO_2_ to a rise in FiO_2_ depends on the amount of venous admixture or pulmonary shunt fraction [8].

As the extracorporeal flow during VV ECMO is in series with native cardiac/pulmonary flows, OCT reliably assesses the pulmonary shunt fraction. However, during veno-arterial (VA) ECMO, extracorporeal flow is in parallel with native cardiac/pulmonary flows, and the OCT results depend on the pulmonary shunt fraction and the relative flow through the extracorporeal circuit. Hence, ECMO flows during VA ECMO may blunt the contribution of native lungs. The use of OCT to assess pulmonary function improvement during VA ECMO is sparsely described in the literature. There is little evidence to support its routine practice or its predictive value for readiness for decannulation. The primary objectives of this study were to evaluate the response of various oxygenation indices to the OCT and to assess the predictive association between oxygenation improvement during the OCT, performed close to decannulation, and survival in neonates supported with VA ECMO for respiratory indications. Secondary objectives included examining the discriminatory ability of oxygenation indices after decannulation for hospital survival and assessing the influence of FiO_2_ on these indices.

## 2. Materials and Methods

In this single-center, retrospective observational study, we reviewed the charts of neonates (≤30 days old) who were placed on VA ECMO for respiratory indications and who underwent an OCT during their ECMO course from 2012 to 2022. This project fell under an IRB-exempt approval (Exempt Approval_PCC Umbrella—Sub Project #16) and was approved on 10 July 2022. This study met the ethical standards of human experimentation, and our research work was in full compliance with the Helsinki Declaration of 1975. We excluded neonates with congenital heart disease. We retrieved demographic, clinical, and outcome data from patients’ charts. We extracted blood gas variables and ventilator parameters before transfer to the hospital (when available), upon arrival at our PICU (Pre-ECMO values), after cannulation, before and during the OCT, and within a day after decannulation (in those who survived the ECMO course). Vasoactive infusion score (VIS) was calculated at the time of cannulation [12,13]. Underlying diagnoses for ECMO support were grouped into MAS, PPHN, CDH, alveolar capillary dysplasia (ACD), and sepsis.

Typically, at our institution, the OCT is performed after the team is weaning ECMO flows and a trial off period is being considered. During the OCT, the ECMO variables (flow and sweep rate) were unchanged. Patient blood gas was obtained with baseline FiO_2_ on the ventilator (typical values were 0.21–0.4). The ventilator FiO_2_ was then increased to 1.0 for at least 15 min without changing any other ventilator parameter. A second blood gas was obtained from the patient to assess the magnitude of the PaO_2_ rise with the OCT. Typically, in our PICU, the majority of these blood gases are drawn from femoral or umbilical arterial lines in these patients; however, we were unable to determine the location of the arterial lines due to the retrospective nature of the study. After completion of the OCT, ventilator FiO_2_ was returned to baseline. If multiple OCTs were performed in a given neonate, the test results closest to decannulation were used for analysis.

### 2.1. Oxygenation Indices

The oxygenation indices, PaO_2_/FiO_2_ ratio (P/F ratio), alveolar-arterial (P(A-a)) gradient, arterial/Alveolar (P(a/A)) ratio, and oxygenation index (OI) were calculated at various time points [14]. The P/F ratio was calculated using the arterial PO_2_ and FiO_2_ on the ventilator [15]. The alveolar gas equation was used to calculate the P(A-a) gradient and the P(a/A) ratio [14]. The OI is calculated using the formula, OI = (Mean airway pressure × FiO_2_ × 100/PaO_2_) [16].

Oxygen tension-based indices are known to be less accurate measures of pulmonary oxygenation capabilities than oxygen content-based indices [14,17]. For accurate measurement of pulmonary shunt fraction, arterial and mixed-venous oxygen contents are required [14]. Accurate measurement of the oxygen content of mixed-venous blood is not feasible in most critically ill children. Several calculations for measuring the shunt fractions without mixed venous oxygen content have been reported in the literature. Of all indices, the Fshunt calculation has been shown to be the most reliable for estimating pulmonary shunt [14,17]. Fshunt was calculated using the formula ([Cc’O_2_ − CaO_2_]/[Cc’O_2_ − CaO_2_ + 3.5 mL/dL]) × 100, where Cc’O_2_ is the oxygen content of pulmonary capillaries, CaO_2_ is the oxygen content of arterial blood, and 3.5 mL/dL was used as the A-v difference [17]. We used an Hb concentration of 10 gm/dL for the calculation of oxygen content. As the sampling sites for mixed venous gas were not clearly identified in our data, we used the calculated Fshunt fraction rather than the pulmonary shunt fraction obtained by the traditional method.

### 2.2. Statistical Analyses

Descriptive statistics are used to summarize demographic variables, ventilator and ECMO parameters, and various oxygenation indices. The categorical variables are presented as percentages, and continuous variables are presented as medians with interquartile ranges (IQRs). The chi-square test was used to compare categorical variables between survivors and non-survivors. Continuous variables between these groups were compared using the Mann–Whitney U test. Paired continuous variables (pre-OCT vs. during OCT) were analyzed using the Wilcoxon signed-rank test.

A linear regression analysis was performed with the increase in PaO_2_ (ΔPaO_2_) during the OCT as the dependent variable. Independent variables included the change in ventilator FiO_2_ (ΔFiO_2_) during the OCT, pre-OCT P/F ratio, ECMO flow (mL/kg/min) during the OCT, and sweep FiO_2_ during the OCT. Results are presented as unstandardized coefficients with 95% confidence intervals (CIs) to indicate effect sizes, and standardized coefficients to facilitate comparison of the relative contribution of each predictor.

Receiver operating characteristic (ROC) curves were constructed to evaluate the discriminatory ability of various oxygenation indices measured after decannulation in predicting mortality. The performance of each index was expressed as the area under the ROC curve (AUC) with corresponding 95% CI. Optimal cutoff values for each variable were determined using the maximum Youden’s index [18].

## 3. Results

We have included 63 neonates on VA ECMO in the study. For post-decannulation analysis, there were 59 neonates (three died before decannulation, and one had no arterial line). Out of 63 neonates, 40 (63.5%) were male infants, 26 (41.9%) belonged to White race, and 13 (20.6%) died during hospitalization. The underlying diagnoses included MAS (33 [52.4%]), sepsis (13 [20.6%]), PPHN (9 [14.3%]), CDH (6 [9.5%]), and ACD (2 [3.2%]).

The demographic characteristics and pre-ECMO indices (P/F, P(A-a), P(a/A), OI, and Fshunt) for all neonates, as well as the comparison between survivors and non-survivors, are shown in Table 1. There were no differences in oxygenation indices prior to cannulation between the two groups (Table 1). Ventilation duration was similar, but survivors had a shorter duration of ECMO support (121 [84–203] vs. 330 [171–420] hours; *p* < 0.001) and a longer hospital stay (60.5 [43.8–88.3] vs. 20.0 [15.5–36.0] days; *p* < 0.001). VIS at the time of cannulation was comparable between survivors and non-survivors (29 [18–43] vs. 32 [18–47]; *p* = 0.799).

At the time of OCT, the median ECMO flow for all patients was 59 (43–78) mL/kg/min, and median airway pressure was 12 (11–14) cmH2O. The median ECMO flow at the time of OCT for survivors and non-survivors was similar: 58 (41–76) and 65 (51–108) ml/kg/min, respectively, and the difference did not approach statistical significance (Table 2). Survivors had a shorter ECMO duration prior to OCT (4.0 [3.0–8.0] vs. 7.0 [4.5–12.5] days; *p* = 0.016) and a shorter interval from OCT to decannulation (0.5 [0.0–1.0] vs. 3.0 [1.5–6.0] days; *p* < 0.001). FiO_2_ (0.31 [0.21–0.46] vs. 0.30 [0.21–0.40]; *p* = 0.384) and PaO_2_ (92 [70–151] vs. 81 [57–102] mmHg; *p* = 0.212) before OCT were similar across groups. PaO_2_ increased significantly with OCT (87 [66–146] vs. 301 [233–371] mmHg; *p* < 0.001). However, the magnitude of PaO_2_ increase, as well as changes in ventilator FiO_2_ and delta PaO_2_ with OCT, did not differ between survivors and non-survivors (Table 2).

All oxygenation indices, both before and during OCT, were comparable between groups (Table 2). Across all neonates, OCT (the increase in ventilator FiO_2_) produced variable effects on oxygenation indices. Specifically, P(A-a) gradient increased (79 [12–165] vs. 359 [288–421] mmHg; *p* < 0.001), P(a/A) ratio decreased (0.54 [0.34–0.87] vs. 0.45 [0.35–0.56]; *p* < 0.001), and Fshunt fraction increased (10.6% [2.6–16.5] vs. 25.3% [21.8–28.6]; *p* < 0.001) with increase in FiO_2_. The only index unaffected by OCT was the P/F ratio (280 [203–467] vs. 301 [233–371]; *p* = 0.239). In linear regression analysis, the magnitude of PaO_2_ increase with OCT was significantly influenced by changes in FiO_2_, the baseline P/F ratio prior to OCT, and ECMO flow at the time of OCT (Table 3).

Following decannulation, survivors demonstrated better oxygenation profiles: higher P/F ratio (162 [119–242] vs. 73 [58–129]; *p* = 0.001) and P(a/A) ratio (0.26 [0.19–0.40] vs. 0.11 [0.09–0.21]; *p* = 0.001), and lower P(A-a) gradient (249 [144–309] vs. 465 [262–522] mmHg; *p* = 0.003), OI (8 [5–12] vs. 20 [10–29]; *p* = 0.002), and Fshunt fraction (22.4% [13.6–26.2] vs. 35.8% [29.8–46.1]; *p* = 0.001) compared with non-survivors (Table 2).

All oxygenation indices measured after decannulation demonstrated fair to moderate discriminatory ability for hospital mortality/survival (Table 4). The optimal cutoff values for each index, derived from the maximum Youden’s index, are summarized in Table 4. Among these, the shunt fraction and P/F ratio showed the strongest performance, with AUCs of 0.853 (95% CI, 0.671–1.0) and 0.844 (95% CI, 0.698–0.991). The optimal cutoffs for shunt fraction and P/F ratio were 28.8% and 111.5, respectively.

We performed an exploratory analysis excluding patients with ACD and CDH, diagnoses associated with substantial mortality, and present the results in Table 5. The overall results are similar to the entire cohort.

## 4. Discussion

In this study of neonates on VA ECMO for respiratory failure, the OCT during ECMO did not differentiate survivors from non-survivors. The observed increase in PaO_2_ during OCT was influenced by the change in ventilator FiO_2_, baseline P/F ratio, and ECMO flow. Of the oxygenation indices evaluated, only the P/F ratio was unaffected by FiO_2_ changes during the OCT. However, oxygenation indices after decannulation showed discriminatory value for predicting hospital survival.

The timing of weaning from ECMO support is critical. Premature weaning may necessitate prolonged post-ECMO support, whereas delayed weaning can result in longer ECMO duration and increased risk of complications, both of which are potentially harmful to patients. In neonates receiving VA ECMO for respiratory support, weaning is usually achieved by gradually reducing ECMO flow while increasing ventilatory support, with decannulation considered once flows are reduced to about 20–40 mL/kg/min. Alternatively, an intermittent trial-off may be performed to assess whether cardiorespiratory function is adequate without ECMO support [19].

A trial-off of VA ECMO is typically undertaken when there is evidence of recovery in both pulmonary (oxygenation and ventilation) and cardiac function, to determine whether ECMO can be safely discontinued. According to ELSO guidelines, discontinuation may be considered when ECMO flows are reduced to 100 mL/min, and the ventilator FiO_2_ is <0.5 with acceptable blood gases [4]. The guidelines also recommend a brief trial-off if the circuit clot burden is low [4]. However, trial-off carries risks, including circuit clotting and cerebral or systemic hemodynamic instability [20,21].

The OCT is a valuable tool for assessing pulmonary oxygenation recovery during VV ECMO [6,7]. In contrast, its utility during VA ECMO is limited because circuit flow runs in parallel with the pulmonary circulation. As demonstrated in our study, the rise in PaO_2_ during OCT was significantly influenced by ECMO flow and the baseline P/F ratio, thereby diminishing the predictive value of this assessment for lung recovery. In neonates on VA ECMO, performing OCTs at progressively lower ECMO flows may provide a more reliable assessment of pulmonary recovery and help define the optimal timing for trial-off. This approach may also reduce the need for repeated premature trial-offs and lower the associated risks.

In neonates with hypoxemic respiratory failure referred to a tertiary care center, an OI greater than 10 predicted the need for ECMO support but did not predict mortality [18]. To our knowledge, no published studies have demonstrated an association between the oxygenation response to the OCT or weaning trials and survival in neonates supported with VA ECMO for respiratory indications. In the present study, the oxygenation response to OCT during ECMO did not predict survival, suggesting that assessment of lung oxygenation function during ECMO may have limited prognostic value. In contrast, oxygenation indices measured after decannulation demonstrated good discriminatory ability for predicting mortality. Although serial OCTs may help identify the optimal timing for a trial-off, oxygenation indices measured during trial-off or after decannulation more accurately reflect the neonate’s ability to maintain adequate oxygenation without extracorporeal support.

Future studies with larger cohorts are needed to validate these findings and to determine whether integrating oxygenation indices during trial-off into clinical decision-making could improve outcomes. However, these indices can be calculated at the bedside using available data during a trial-off period and may provide further context for the patient’s native pulmonary function. This, in turn, could influence decannulation decisions. Furthermore, exploring their association not only with survival but also with other outcomes such as hospital length of stay, longer-term respiratory morbidity, and neurodevelopmental outcomes may be valuable.

### 4.1. The Effect of FiO_2_ on Oxygenation Indices

Several indices have been used to assess the oxygenation function of the native lung [14,22,23]. The utility and limitations of tension-based oxygenation indices are well described in the literature [24]. These indices are generally unreliable for estimating pulmonary shunt because of their non-linear response to changes in FiO_2_ [25,26]. Among them, the P/F ratio demonstrated the most stability in adults with ARDS when FiO_2_ was between 0.5 and 1.0 [24,26].

In neonates, however, oxygenation indices, including the P/F ratio, P(a/A) ratio, and OI, were strongly affected by FiO_2_ adjustments, with the degree of change proportional to FiO_2_ levels [27]. Of these, the P(a/A) ratio performed relatively better [27]. In our study, the P/F ratio remained unchanged during the OCT, whereas all other indices varied significantly with increasing FiO_2_. For neonates with hypoxemic respiratory failure, OI is commonly used to classify severity because it incorporates mean airway pressure [16]. Since the P/F ratio is not routinely applied in this population, its utility for assessing neonatal hypoxemic respiratory failure requires further evaluation.

Measured shunt fractions are typically FiO_2_-independent and more reliable. In our study, the calculated Fshunt fraction also changed with FiO_2_. This may be explained by our use of assumed fixed values for A–V difference and hemoglobin concentration, which could have influenced the accuracy of shunt estimation.

### 4.2. Limitations

There are several limitations to this study. First, this is a retrospective study of a single center without a priori defined criteria for when to perform the OCT (clinician-dependent). Second, we were unable to analyze the OCT at different, decremental ECMO flows, which may have helped delineate its value. Third, the response to OCT could be influenced by the distance between the ECMO arterial cannula and the sampling arterial cannula due to native/extracorporeal flow mixing, and we did not reliably identify this in our study. Fourth, we did not evaluate various oxygenation indices during trial-off periods, which could have given further information on their relationship with outcomes and the trial-off period. Fifth, our F-shunt results should be interpreted cautiously and treated as exploratory, as we assumed a hemoglobin concentration of 10 g/dL and a fixed arteriovenous content of 3.5 mL/dL in our calculations. Finally, this was a single-center study that limits the number of patients we could have included.

## 5. Conclusions

In conclusion, these results indicate that the OCT response during VA ECMO was not associated with hospital survival and was significantly influenced by ECMO flow. Whether OCT performed at standardized low ECMO flow or during trial-off can assist decannulation decisions remains unanswered. Clinical decisions regarding decannulation should not rely on an isolated OCT during VA ECMO.

## Figures and Tables

**Table 1:** Comparison of demographic and clinical variables in survivors and deceased neonates who were on VA ECMO.

Variable	All (n = 63)	Survivors (n = 50)	Deceased (n = 13)	Significance
Age at cannulation (Days)	2 (1–4)	2 (1–4)	1 (0.5–7.5)	*p* = 0.405
Male (%)	40 (63.5)	33 (66.0)	7 (53.8)	*p* = 0.417
Weight (kgs)	3.35 (2.94–3.64)	3.38 (3.07–3.67)	3.01 (2.45–3.32)	*p* = 0.027
ACD ^a^ and CDH ^b^ (%)	8 (12.7)	1 (2)	7 (53.8)	*p* < 0.001 ^c^
VIS ^d^ at cannulation	30 (18–44)	29 (18–43)	32 (18–47)	*p* = 0.799
P/F ^e^ at cannulation	37 (31–45)	38 (31–45)	36 (30–45)	*p* = 0.825
P(A-a) ^f^ at cannulation (mmHg)	611 (590–624)	612 (594–624)	593 (568–618)	*p* = 0.096
P(a/A) ^g^ at cannulation	0.058 (0.047–0.069)	0.058 (0.046–0.068)	0.053 (0.048–0.075)	*p* = 0.905
OI ^h^ at cannulation	44 (36–62)	47 (37–64)	42 (32–54)	*p* = 0.304
Fshunt ^i^ at cannulation (%)	54.4 (46.9–59.9)	54.0 (46.5–59.9)	54.7 (51.9–60.0)	*p* = 0.634
ECMO duration (Hours)	146 (91–242)	121 (84–203)	330 (171–420)	*p* < 0.001
Duration of ventilation (days)	25.0 (17.0–32.0)	25.0 (17.8–31.3)	20.0 (15.5–36.0)	*p* = 0.552
Hospital length of stay (days)	50.0 (36.0–80.0)	60.5 (43.8–88.3)	20.0 (15.5–36.0)	*p* < 0.001

Continuous variables are presented as median (Interquartile range); ^a^. ACD = alveolar capillary dysplasia; ^b^. CDH = congenital diaphragmatic hernia; ^c^. Fisher’s Exact test; ^d^. VIS = vasoactive infusion score; ^e^. P/F = PaO_2_ and FiO_2_ ratio; ^f^. P(A-a) = alveolar arterial PO_2_ gradient; ^g^. P(a/A) = arterial and alveolar PO_2_ ratio; ^h^. OI = oxygenation index; ^i^. Fshunt = shunt fraction.

**Table 2:** Oxygenation and ECMO variables with the oxygen challenge test in survivors and deceased neonates who were on VA ECMO.

Variable	All (n = 63)	Survivors (n = 50)	Deceased (n = 13)	Significance ^b^	Paired analysis ^c^
ECMO flow at OCT ^a^ (ml/kg/min)	59 (43–78)	58 (41–76)	65 (51–108)	*p* = 0.080	
Time: cannulation to OCT (days)	5.0 (3.0–8.0)	4.0 (3.0–8.0)	7.0 (4.5–12.5)	*p* = 0.016	
Time: OCT to decannulation (days)	1.0 (0.0–2.0)	0.5 (0.00–1.0)	3.0 (1.5–6.0)	*p* < 0.001	
FiO_2_ (Ventilator)-Pre-OCT	0.30 (0.21–0.45)	0.31 (0.21–0.46)	0.30 (0.21–0.40)	*p* = 0.384	
PaO_2_—Pre-OCT	87 (66–146)	92 (70–151)	81 (57–102)	*p* = 0.212	<0.001
PaO_2_—with OCT	301 (233–371)	292 (247–359)	301 (219–408)	*p* = 0.852	
Delta FiO_2_ with OCT	0.70 (0.55–0.79)	0.69 (0.54–0.79)	0.70 (0.60–0.79)	*p* = 0.384	
Delta PaO_2_ with OCT	175 (131–248)	176 (131–237)	168 (128–271)	*p* = 0.740	
P/F ^d^—Pre-OCT	280 (203–467)	299 (202–470)	240 (206–452)	*p* = 0.481	0.239
P/F—with OCT	301 (233–371)	292 (247–359)	301 (219–408)	*p* = 0.852	
P(A-a) ^e^—Pre-OCT	79 (12–165)	78 (14–167)	82 (0–161)	*p* = 0.799	<0.001
P(A-a)—with OCT	359 (288–421)	367 (297–422)	338 (256–439)	*p* = 0.865	
P(a/A) ^f^—Pre-OCT	0.54 (0.34–0.87)	0.55 (0.35–0.86)	0.46 (0.34–0.99)	*p* = 0.721	<0.001
P(a/A)—with OCT	0.45 (0.35–0.56)	0.44 (0.37–0.55)	0.47 (0.33–0.61)	*p* = 0.932	
Fshunt ^g^—Pre-OCT	10.6 (2.6–16.5)	10.2 (2.3–16.2)	13.9 (5.7–17.4)	0.760	<0.001
Fshunt—with OCT	25.3 (21.8–28.6)	25.1 (21.8–28.1)	26.4 (21.0–31.3)	0.316	
P/F after decannulation	145 (110–222)	162 (119–242)	73 (58–129)	*p* = 0.001	
P(A-a) after decannulation	264 (149–346)	249 (144–309)	465 (262–522)	*p* = 0.003	
P(a/A) after decannulation	0.23 (0.17–0.38)	0.26 (0.19–0.40)	0.11 (0.09–0.21)	*p* = 0.001	
OI ^h^ after decannulation	10 (5.3–12.8)	8 (5–12)	20 (10–29)	*p* = 0.002	
Fshunt after decannulation	23.0 (14.1–28.8)	22.4 (13.6–26.2)	35.8 (29.8–46.1)	0.001	

Continuous variables are presented as median (Interquartile range); ^a^. OCT = oxygen challenge test; ^b^. Comparison between survivors and deceased; ^c^. Paired comparison between pre- and during OCT; ^d^. P/F = PaO_2_ and FiO_2_ ratio; ^e^. P(A-a) = alveolar-arterial PO_2_ gradient; ^f^. P(a/A) = arterial and alveolar PO_2_ ratio; ^g^. Fshunt = shunt fraction; ^h^. OI = oxygenation index.

**Table 3:** Variables influencing the change in PaO_2_ with the oxygen challenge test.

Variable	Unstandardized Coefficients (B)	Significance	Standardized Coefficients (Beta)	VIF ^a^
(Constant)	−20.0 (−190.7–150.6)	0.81		
Delta ventilator FiO_2_ with OCT ^b^	267.84 (89.5–446.2)	0.004	0.38	1.35
P/F ^c^ before OCT	−0.16 (−0.25–−0.07)	0.001	−0.44	1.35
ECMO Flow (mL per/kg/min) with OCT	1.34 (0.53–2.14)	0.002	0.37	1.02
FiO_2_ sweep with OCT	19.2 (−109.7–148.1)	0.77	0.03	1.02

^a^. VIF = Variance Inflation Factor; ^b^. OCT = oxygen challenge test; ^c^. P/F = PaO_2_ and FiO_2_ ratio.

**Table 4:** Receiver Operating Curves for various oxygenation indices after decannulation of neonates on VA ECMO.

Oxygenation Index	Outcome Variable ^a^	AUC (95% CI)	*p*-Value	Cut-Off Value at Maximum Youden Index
P/F ^b^	Survival	0.844 (0.698–0.991)	<0.001	111.5
OI ^c^	Mortality	0.828 (0.671–0.984)	<0.001	12.6
P(A-a) ^d^	Mortality	0.778 (0.587–0.969)	0.004	442.5 mmHg
P(a/A) ^e^	Survival	0.842 (0.687–0.997)	<0.001	0.173
Fshunt ^f^	Mortality	0.853 (0.671–1.0)	<0.001	28.8%

^a^. Higher values of the oxygenation index are associated with the outcome variable; ^b^. P/F = PaO_2_ and FiO_2_ ratio; ^c^. OI = oxygenation index; ^d^. P(A-a) = alveolar arterial PO_2_ gradient; ^e^. P(a/A) = arterial and alveolar PO_2_ ratio; ^f^. Fshunt = shunt fraction.

**Table 5:** Oxygenation and ECMO variables with the oxygen challenge test in survivors and deceased neonates who were on VA ECMO and without a diagnosis of ACD or CDH.

Variable	All (n = 55)	Survivors (n = 49)	Deceased (n = 6)	Significance ^b^	Paired Analysis ^c^
ECMO flow at OCT ^a^ (ml/kg/min)	59 (41–78)	59 (41–75)	87 (47–115)	*p* = 0.196	
FiO_2_ (Ventilator)-Pre-OCT	0.32 (0.21–0.45)	0.32 (0.21–0.48)	0.35 (0.21–0.43)	*p* = 0.864	
PaO_2_—Pre-OCT	82 (65–146)	96 (69–156)	87 (50–164)	*p* = 0.571	<0.001
PaO_2_—with OCT	305 (252–381)	301 (252–360)	313 (178–469)	*p* = 0.948	
Delta FiO_2_ with OCT	0.68 (0.55–0.79)	0.68 (0.53–0.79)	0.65 (0.58–0.79)	*p* = 0.722	
Delta PaO_2_ with OCT	175 (129–247)	177 (130–241)	152 (107–327)	*p* = 0.864	
P/F ^d^—Pre-OCT	267 (178–410)	292 (201–471)	229 (178–612)	*p* = 0.645	0.218
P/F—with OCT	313 (252–381)	301 (252–360)	313 (178–469)	*p* = 0.948	
P(A-a) ^e^—Pre-OCT	79 (17–179)	79 (10–169)	93 (12–197)	*p* = 1.000	0.218
P(A-a)—with OCT	339 (283–412)	359 (294–420)	348 (186–482)	*p* = 0.906	
P(a/A) ^f^—Pre-OCT	0.51 (0.30–0.82)	0.54 (0.34–0.89)	0.49 (0.29–1.18)	*p* = 0.844	0.018
P(a/A)—with OCT	0.47 (0.38–0.57)	0.45 (0.38–0.55)	0.47 (0.27–0.71)	*p* = 0.948	
Fshunt ^g^—Pre-OCT	10.6 (2.4–18.8)	10.6 (2.2–16.3)	16.1 (0.3–17.6)	0.645	<0.001
Fshunt—with OCT	25.1 (20.6–28.8)	25.1 (21.7–28.2)	28.7 (18.9–31.6)	0.451	
P/F after decannulation ^i^	152 (113–237)	160 (120–240)	73 (71–92)	*p* = 0.008	
P(A-a) after decannulation ^i^	258 (149–340)	251 (149–305)	468 (407–537)	*p* = 0.005	
P(a/A) after decannulation ^i^	0.24 (0.18–0.40)	0.26 (0.19–0.40)	0.11 (0.11–0.14)	*p* = 0.004	
OI ^h^ after decannulation ^i^	10 (5.3–12.6)	8.4 (5.3–11.7)	24.8 (18.7–26.2)	*p* = 0.006	
Fshunt after decannulation ^i^	22.7 (13.7–27.4)	22.5 (13.6–26.2)	38.7 (37.3–43.2)	<0.001	

Continuous variables are presented as median (Interquartile range); ^a^. OCT = oxygen challenge test; ^b^. Comparison between survivors and deceased; ^c^. Paired comparison between pre- and during OCT; ^d^. P/F = PaO_2_ and FiO_2_ ratio; ^e^. P(A-a) = alveolar-arterial PO_2_ gradient; ^f^. P(a/A) = arterial and alveolar PO_2_ ratio; ^g^. Fshunt = shunt fraction; ^h^. OI = oxygenation index; ^i^. the total n = 52, with 3 died.

## Data Availability

Hospital-specific data; Data not available for privacy, legal, and data protection reasons.
